# The hidden inequality: the disparities in the quality of daily use masks associated with family economic status

**DOI:** 10.3389/fpubh.2023.1163428

**Published:** 2023-06-16

**Authors:** Lei Hua, Ying Wang, Bijuan Mo, Zuqi Guo, Yulei Wang, Zexuan Su, Minqi Huang, Han Chen, Xiaowen Ma, Jiaxin Xie, Mengxian Luo

**Affiliations:** ^1^School of Public Administration, Nanfang College · Guangzhou, Guangzhou, China; ^2^School of Government, Sun Yat-sen University, Guangzhou, China; ^3^School of Public Policy and Administration, Chongqing University, Chongqing, China; ^4^School of Foreign Language, Hunan University of Technology and Business, Changsha, Hunan, China

**Keywords:** COVID-19, the quality of masks, family economic status, hidden socioeconomic inequality, particle filtration efficiency, personal protection equipment

## Abstract

Wearing high-quality masks plays a critical role in reducing COVID-19 transmission. However, no study has investigated socioeconomic inequality in the quality of masks. Addressing this gap, this paper explored the relationships between mask’s quality and family economic status. The cross-sectional survey was conducted in two Chinese universities by distributing structured questionnaires to assess participants’ characteristics including family economic status, and meanwhile collecting their masks to evaluate the quality by measuring particle filtration efficiency. The valid responses were obtained from 912 students with mean age of 19.556 ± 1.453  years and were analyzed by using fractional or binary logistic regression. Three main findings were presented. First, inequality existed in the quality of masks. 36.07% of students were using unqualified masks with average filtration efficiency of 0.795 ± 0.119, which was much lower than China’s national standard (0.9). Of those masks with identified production date, 11.43% were manufactured during COVID-19 outbreak when market was flooded with counterfeit production, and thus were of poor quality with average filtration efficiency of 0.819 ± 0.152. Second, better family economic status was associated with better masks’ filtration efficiency and greater probability of using qualified masks. Third, students with better family economic status tend to use masks with individual packaging, and unique patterns and special designs, which may lead to inequality on a psychological level. Our analysis reveals the hidden socioeconomic inequality that exist behind cheap masks. In facing the challenges of future emerging infectious diseases, it is important to address the inequity to ensure equal access to affordable qualified personal protection equipment.

## 1. Introduction

Coronavirus disease 2019 (COVID-19), which was caused by the novel severe acute respiratory syndrome coronavirus 2 (SARS-CoV-2), was first identified in Wuhan City, Hubei Province, China, in late December 2019 and has spread rapidly around the world (1). As of 10 May 2023, more than 765 million people were infected, and more than 6.9 million people died as a result of COVID-19 infection, posing a great risk to public health (2, 3). Evidence demonstrates that the virus can infect through airborne transmission routes when an infected person ‘exhales, speaks, shouts, sings, sneezes, or coughs’ (4, 5), which makes the wearing of high-quality masks of particular importance in reducing COVID-19 transmission among the public (3, 4, 6–13). Although there is now substantial evidence for the effectiveness of wearing masks in mitigating the spread of COVID-19 (14–24), surprisingly little research attention has been paid to mask equity related issues such as the inequity in mask use (4) and the mask-wearing behavior associated with racial and ethnic disparities (25, 26). To date, no study has been done to investigate the disparities in the quality of masks associated with socioeconomic status.

When something appears to be so inexpensive that anyone can afford it (the mask costed about ¥0.20–¥0.50 a piece when we conducted this study) (27), people often ignore the inequality behind the product and may fail to recognize that such inequality exists, plus the fact that all masks look similar and it is hardly to tell the quality difference by naked eye. However, we believe that the invisible but serious inequity in the quality of daily-use masks exists. Because in our assumption, the rich (the monthly living expenses≥¥2000 in this study) may value the quality of the masks and do not care much about the price, while the poor (the monthly living expenses<¥1000 in this study) may be more concerned about money and tend to buy the cheapest masks among the already cheaper ones, and therefore use the low-quality or even unqualified masks. The invisible difference in mask quality can only be measured by using professional equipment to test their particle filtration efficiency. This kind of invisible inequality can lead to serious public health problems because the unqualified masks that the poor use cannot provide effective protection like authentic ones and may further increase the social health risk during pandemic (3).

**Figure 1:**
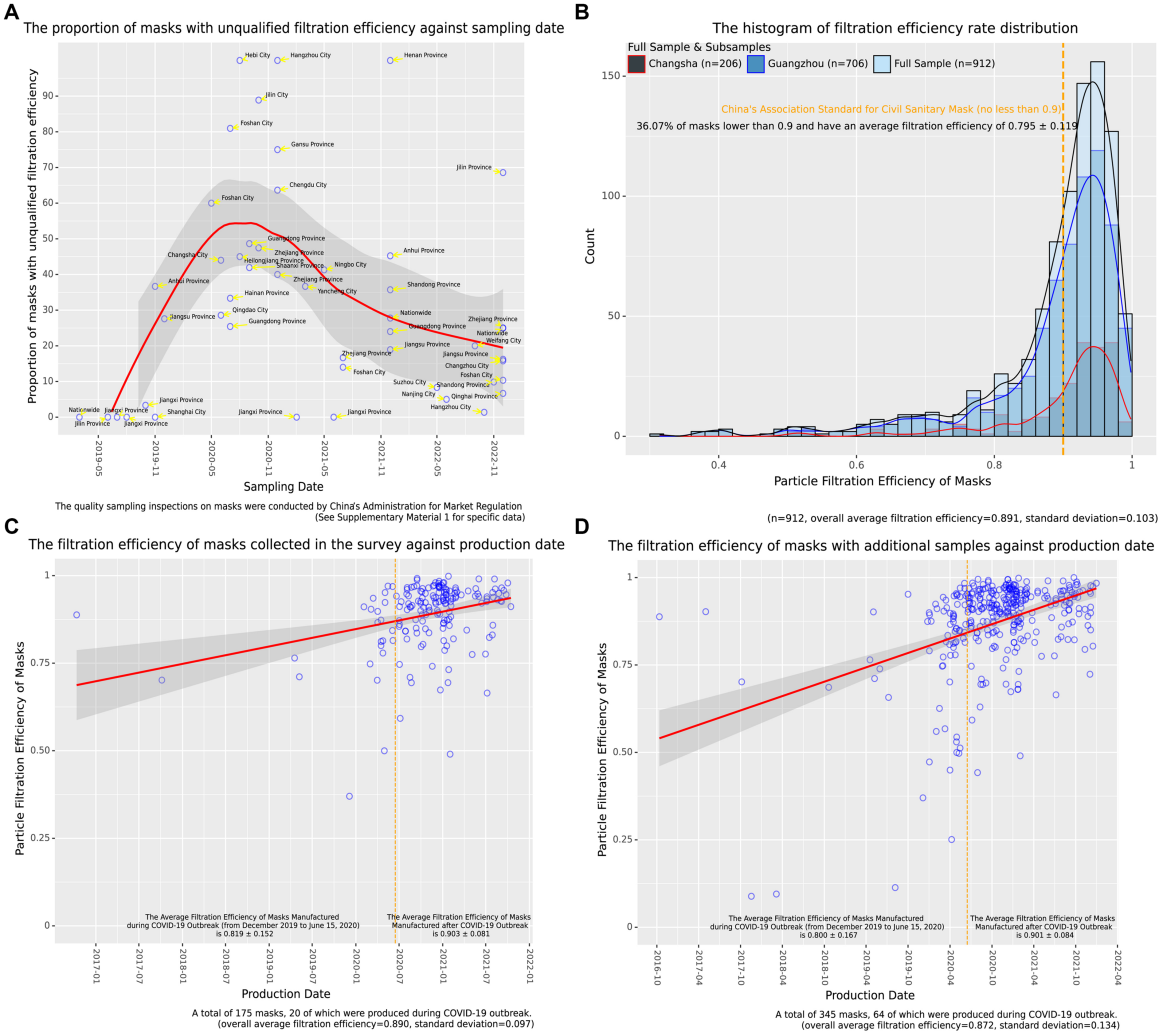
**(A)** The proportion of masks with unqualified filtration efficiency against sampling date. **(B)** The histogram of filtration efficiency rate distribution. **(C)** The filtration efficiency of masks collected in the survey against production date. **(D)** The filtration efficiency of masks with additional samples against production date.

In fact, the poor-quality masks circulating in the Chinese market has been a very serious problem since the COVID-19 outbreak. The poor filtration efficiency was the most important factor in the unqualified masks (28). By collecting the results of quality sampling inspection on masks conducted by China’s Administration for Market Regulation, we plotted the scatter of proportion of masks with unqualified filtration efficiency against sampling date, as shown in Figure 1A. As we can see from Figure 1A, the unqualified rate of masks before 2020 was very low, for example, in the national sampling of masks, there was no case of failure due to filtration performance. However, in the year 2020 when the COVID-19 pandemic broke out, the quality of masks available in China’s market became extremely poor, with the unqualified rate of mask filtration efficiency in various regions exceeding 40%, and even reaching 100%. After that, although the unqualified rate declined over time, by the end of 2021 when we conducted our research, the unqualified rate of masks in the national sampling inspection still reached 27.78%, and even at the end of 2022, the nationwide unqualified rate remained as high as 25%. To combat the low-quality masks and reduce the public health risk, the government may inspect mask quality in the market, however, which may not be always effective (3). As low-cost and low-quality masks circulate in the market, the quality of masks that people use daily may vary greatly. It is therefore important to find out whether the hidden socioeconomic inequality exists in mask quality and to make targeted policy recommendations.

This study aims to investigate the relationships between the quality of masks and family economic status. To achieve that goal, we distributed questionnaires to assess the participants’ characteristics including family economic status, and collected the participants’ new masks for daily use to measure the quality. By conducting fractional logistic regression and binary logistic regression with family economic status as core independent variable and the quality of masks as dependent variable, this paper contributes to filling the above-mentioned research gap by providing empirical answers to the disparity in the quality of masks and its relationship with family economic status. Family economic status was captured through examining the living expenses obtained from families. Quality of masks were measured from three aspects: the quality of filtration performance (including particle filtration efficiency, and whether the particle filtration efficiency is qualified), the quality of hygiene and cleanliness (whether the mask is individually wrapped) and the quality of aesthetics and design (whether the mask has the unique pattern or special design). We hypothesize that greater family economic status increases the probability of using masks with better particle filtration efficiency, the probability of using qualified masks, and the probability of using masks with individual packaging, and unique patterns and special designs.

## 2. Methods

### 2.1. Study sample and data collection

According to the previous study, those who were highly educated were more likely to wear masks (26). Thus, college students in China were chosen as the research subjects of the study. However, due to the strict control over student registration in China, the entire list of students in any university is not accessible for sampling without official permission (29). Therefore, convenience sampling was used to recruit the participants. The survey was conducted from 10 to 22 November 2021 in two Chinese universities where the researchers are currently working, one in South China’s Guangzhou and another in Central China’s Changsha. We randomly selected one college in each of the two universities, and then conducted questionnaires and collected masks from students in the selected college during their classes. Course teachers and counselors helped us to informed students in advance about the content, time and the specific place of the survey and reminded the participators that they needed to bring a new daily-use mask. All the participants were informed that their participation was voluntary, anonymous, and confidentiality was assured (1). Since the questionnaires were anonymous, written informed consent was not obtained (30–32), and the return of the completed questionnaire and a new daily-use mask implied informed consent (1, 33–37). Each participant received a ¥1 honorarium and two packages of snacks for participating. Only participants who completed the questionnaires and submitted new masks were included in our analyses. A total of 912 qualified participants with mean age of 19.556 ± 1.453 years completed the survey; of those, 706 were recruited from Guangzhou and 206 were from Changsha. Unfortunately, due to the sudden outbreak of COVID-19 in Changsha in late October 2021 and the following strict pandemic control measures, we could hardly enlarge our sample size in Changsha.

### 2.2. Measurement

#### 2.2.1. Dependent variable: the quality of masks

**Figure 2:**
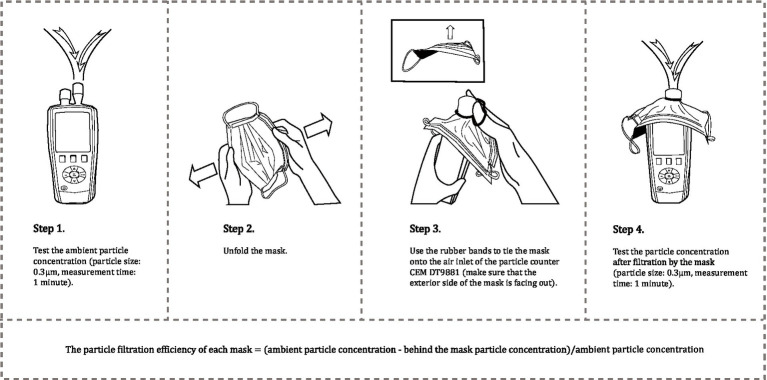
The procedure of testing the particle filtration efficiency of masks (calculation of particle filtration efficiency = (ambient particle concentration−behind the mask particle concentration) / ambient particle concentration).

The particle filtration efficiency of masks, which is a continuous dependent variable that most directly measures the quality of the masks (38), is the main interest of this study. The particle filtration efficiency of each mask was determined by measuring the difference in the number of particles before and after filtration by the mask (39) (see Figure 2), and precisely was calculated as (ambient particle concentration − behind the mask particle concentration) /ambient particle concentration (38, 40–43). From the calculated formula, we can see that the value of this continuous dependent variable is greater than or equal to 0 and less than or equal to 1 (44). The testing was performed with the particle counter CEM DT9881 (45–48), which can measure particles with a minimum size of 0.3 μm at a sampling flow rate of 2.83 L/min (49, 50). Taking into consideration that the Coronavirus is mainly spread through particles with diameters of approximately 0.3 μm or more extensive (39, 51, 52). Meanwhile, 0.3 μm aerosol size has been adopted as the particle standard of the NIOSH N95 filter certification test (53) and has been widely used as the index to test masks in previous studies (43, 54–58). The filtration efficiency at the most penetrating particle size of 0.3 μm was therefore used to calculate the mask quality. Then the new masks collected from the survey were tested to measure their filtration performance. The total testing time for each mask was about 3 min (59).

To ensure that our major findings are robust, this study also divided the particle filtration efficiency of masks into two categories based on China’s association standard for the civil sanitary mask (≥90% filtration efficiency for 0.3 μm particles): unqualified (<0.9) and qualified (≥0.9), which were labeled 0 and 1, respectively.

In addition, this paper also explores the relationships between family economic status and two binary variables that reflect the quality of hygiene and cleanliness (whether the mask is individually wrapped), and the quality of aesthetics and design (whether the mask has the fashionable and attractive unique pattern or special design) (see the Supplementary material 2). The two variables are chosen because the issue of hygiene and cleanliness in mask wearing is also a key step to lower the transmission during pandemic (60), and mask’s appearance has become an important factor in people’s choice of buying or using masks (61, 62). For the two variables, yes was coded 1, and no was coded 0 (see Table 1).

**Table 1:** Descriptive statistics of full sample and sub-samples.

	Full sample	Samples collected in Guangzhou	Samples collected in Changsha
Number	(*n* = 912)	(*n* = 706)	(*n* = 206)
**Quality of filtration performance: particle filtration efficiency of the mask**	0.891 ± 0.103	0.888 ± 0.106	0.900 ± 0.095
**Quality of filtration performance: whether the particle filtration efficiency of the mask is qualified** (**≥0.9**)			
Unqualified = 0	329 (36.07)	266 (37.68)	63 (30.58)
Qualified = 1	583 (63.93)	440 (62.32)	143 (69.42)
**Quality of hygiene and cleanliness: whether the mask is individually wrapped**			
No = 0	574 (62.94)	412 (58.36)	162 (78.64)
Yes = 1	338 (37.06)	294 (41.64)	44 (21.36)
**Quality of aesthetics and design: whether the mask has the unique pattern or special design**			
No = 0	802 (87.94)	617 (87.39)	185 (89.81)
Yes = 1	110 (12.06)	89 (12.61)	21 (10.19)
**Family economic status: living expenses obtained from family**			
Poor (less than ¥1000) = 1	157 (17.21)	128 (18.13)	29 (14.08)
Average (¥1000 to less than ¥2000) = 2	626 (68.64)	477 (67.56)	149 (72.33)
Rich (¥2000 or more) = 3	129 (14.14)	101 (14.31)	28 (13.59)
**Gender**			
Male = 0	199 (21.82)	179 (25.35)	20 (9.71)
Female = 1	713 (78.18)	527 (74.65)	186 (90.29)
**Age**	19.556 ± 1.453	19.725 ± 1.332	18.976 ± 1.686
**Nationality**			
Han = 0	883 (96.82)	691 (97.88)	192 (93.20)
Minority = 1	29 (3.18)	15 (2.12)	14 (6.80)
**Religious belief**			
No = 0	863 (94.63)	665 (94.19)	198 (96.12)
Yes = 1	49 (5.37)	41 (5.81)	8 (3.88)
**Political background**			
The masses = 1	117 (12.83)	105 (14.87)	12 (5.83)
CCYL member = 2	694 (76.10)	562 (79.60)	132 (64.08)
Democratic parties and non-partisan = 3	0 (0.00)	0 (0.00)	0 (0.00)
CPC member = 4	101 (11.07)	39 (5.52)	62 (30.10)
**BMI**	20.160 ± 3.022	20.142 ± 3.156	20.221 ± 2.518
**Number of siblings**	1.201 ± 1.090	1.238 ± 1.166	1.073 ± 0.765
**Whether the participant’s parent is alive**			
One or both parents died = 0	37 (4.06)	23 (3.26)	14 (6.80)
Both parents alive = 1	875 (95.94)	683 (96.74)	192 (93.20)

Data are expressed as number (percentage) for categorical variables and mean ± standard deviation for continuous variables.

#### 2.2.2. Core independent variable: family economic status

This paper uses living expenses obtained from families to capture the family economic status of subjects (29). In China, it is common for parents to provide financial support for their children’s education according to their own economic capabilities (29, 63). Moreover, for the full-time college students in China, family is the main source of fees and expenses (29, 63, 64). Thus, the living expenses obtained from the family can provide a valid proxy for the economic status of the family (29). The core independent variable in this paper is the average, estimated, monthly, living expenses obtained from the parents and other family members, exclusive of tuition or fees (29).

According to previous survey, the median monthly living expenses of Chinese college students in 2020 was ¥1516 (65), and the average monthly living expenses of college students in Guangdong Province and Hunan Province, where our survey was conducted, were ¥1612 and ¥1323, respectively, in the year of 2019 (66). Therefore, this study defines students with monthly living expenses below ¥1000 as poor, those with incomes between ¥1000 and ¥2000 as average, and those with incomes above ¥2000 as rich. The living expenses were therefore divided into 3 ordered levels, coded as 1 to 3, representing poor (<¥1000), average (¥1000 – < ¥2000), and rich (≥¥2000), respectively (see Table 1).

#### 2.2.3. Control variables

As the mask choosing and purchasing behavior is a matter of personal freedom (67), and, at the end of 2021, the supply of masks in the Chinese market was sufficient, this study considered individual-level factors rather than macro-level factors as the control variables. By following the previous literature, the personal socio-demographic characteristics such as gender (6, 17, 19, 68–70), age (6, 17, 68–70), nationality (17, 26, 71), religious belief (72, 73), political background (67, 73) and body mass index (BMI) (68, 70, 74), and family characteristics such as the number of siblings (1, 72) and parental status (whether the participant’s parent is alive) (71, 72, 75) were accessed by questionnaires and included as the control variables. The values assigned to the control variables are listed in Table 1.

#### 2.2.4. Other variables

Additionally, the manufacture date of the masks was also recorded (if available), which can help us to understand the trends in mask quality over time. In our survey, a total of 175 masks with identified production dates were obtained.

### 2.3. Statistical analysis methods

This paper used fractional logistic regression for the continuous dependent variable (particle filtration efficiency of masks) (44) and binary logistic regression for binary dependent variables (whether the particle filtration efficiency of the mask is qualified, whether the mask is individually wrapped, and whether the mask has the unique pattern or special design).

**Figure 3:**
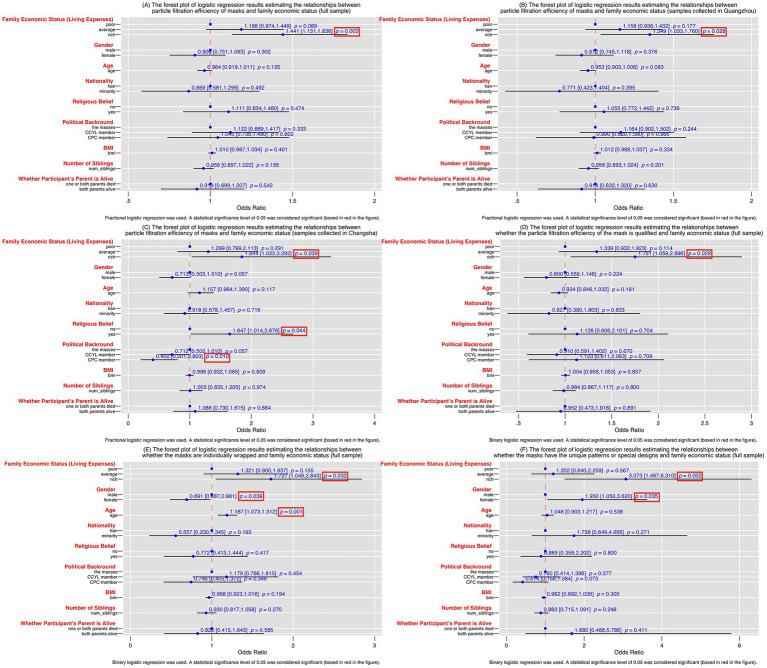
**(A)** The forest plot of logistic regression results estimating the relationships between particle filtration efficiency of masks and family economic status (full sample). **(B)** The forest plot of logistic regression results estimating the relationships between particle filtration efficiency of masks and family economic status (samples collected in Guangzhou). **(C)** The forest plot of logistic regression results estimating the relationships between particle filtration efficiency of masks and family economic status (samples collected in Changsha). **(D)** The forest plot of logistic regression results estimating the relationships between whether the particle filtration efficiency of the mask is qualified and family economic status (full sample). **(E)** The forest plot of logistic regression results estimating the relationships between whether the masks are individually wrapped and family economic status (full sample). **(F)** The forest plot of logistic regression results estimating the relationships between whether the masks have the unique patterns or special designs and family economic status (full sample).

To investigate the relationships between family economic status and the quality of masks, the fractional logistic regression model was first conducted with particle filtration efficiency of masks as the dependent variable and students’ living expenses obtained from family as the core independent variable (see Figure 3A). Then we conducted sensitivity tests in the fractional logistic regression models by dividing the full sample into separate subsamples based on the collection location, to ensure that our major findings are robust (see Table 1 and Figures 3B,C). By dividing the particle filtration efficiency of masks into unqualified and qualified, this paper also used binary logistic regression models to examine associations between whether the particle filtration efficiency of the mask is qualified and students’ living expenses (see Figure 3D). The binary logistic regression model was also conducted with two binary variables that reflect the quality of hygiene and cleanliness and the quality of aesthetics and design as the dependent variables (see Figures 3E,F). All analysis was conducted by using Stata 17 and the statistical significance level was taken as 0.05 in all tests (76).

## 3. Results

### 3.1. Descriptive statistics: the disparities in the quality of masks

The statistical characteristics of the samples are shown in Table 1. Here, we focus on the masks’ particle filtration efficiency. Overall, the masks that used daily by Chinese college students had an average filtration efficiency rate of 0.891 ± 0.103 for 0.3 μm size particles. Despite the difference in sample size, there was little difference in the average efficiency between the masks collected in Guangzhou and Changsha. By plotting the histogram of filtration efficiency rate distribution (see Figure 1B), we observed that the samples collected in two different cities had very similar distribution patterns. In terms of the proportion of unqualified masks, there were a significant number (36.07%) of students using masks with filtration efficiency lower than the national standard and the average filtration efficiency of their masks was only 0.795 ± 0.119.

The inequality can also be reflected in the change in mask quality over time. Figure 1C shows that, of the masks with a clear date of manufacture, about 11.43% were produced during the COVID-19 outbreak (from December 2019 to June 15, 2020) when the facemasks were in severe shortage in China and were therefore of poor quality with an average filtration efficiency of 0.819 ± 0.152 (77), compared to 0.903 ± 0.081 for masks produced after June 15 2020. To make our result more accurate, we obtained 170 additional unused masks with production dates from different sources such as markets and friends. The filtration efficiency of masks including additional samples against the production date was plotted in Figure 1D, also demonstrating that the masks manufactured during the COVID-19 outbreak were generally of relatively low quality with an average filtration efficiency of 0.800 ± 0.167.

### 3.2. The relationships between particle filtration efficiency of masks and family economic status

From Figure 3A, we can clearly see that compared with the students from poor families, the odds ratios of the other two family economic levels were both higher than 1 and became higher with improvements in family economic status. Similar increasing trends were observed not only in the full sample but also in the sub-samples divided by geography (Figure 3B and Figure 3C). Specifically, we take the fractional regression results of the full sample (Figure 3A) as an example: the odds ratios for average and rich were 1.188 and 1.441, respectively, when compared with the poor. We can also observe the increase in odds ratios with improvements in the family economic status from Figure 3D. The odds ratios were 1.339 and 1.751, corresponding to the levels of family economic status from average to rich. Although the odds ratios were increasing with family economic status, only the level of rich family was always statistically significant (*p* < 0.05) in all four estimated models, suggesting that the gap in mask quality between the poor and the rich was particularly striking.

It is noteworthy that, the regression of Changsha sample had some unusual results, that is, students with religious belief had a significant (*p* = 0.044) higher odds ratio (1.647) than those without, while the CPC membership had a significant (*p* = 0.010) lower odds ratio (0.402) than others.

### 3.3. The relationships between the quality of hygiene and cleanliness, the quality of aesthetics and design, and family economic status, respectively

From Figures 3E,F, we can also clearly see that compared with the students from poor families, the odds ratios of the other two family economic levels were both higher than 1. And with advancing family economic status, the odds ratio showed increasing trends, especially in the rich level in Figure 3F, where the odds ratio reached 3.073 (*p* = 0.002). Also, similar to the foregoing analysis, the most significant difference remained between the poor and the rich (*p* < 0.05).

Notably, the variable of gender was also significant in both models. Females had a significant (*p* = 0.039) lower odds ratio (0.691) of using individually wrapped masks compared to males, whereas females had a significant (*p* = 0.035) higher odds ratio (1.950) than males in using masks that having unique pattern or special design. In addition, the variable of age in Figure 3E also had a significant odds ratio of 1.187.

## 4. Discussion

We discuss our finding as follows. First, inequality existed in the quality of masks for daily use. The masks that used daily by Chinese college students had an average filtration efficiency rate of 0.891 ± 0.103 for 0.3 μm size particles, which was a little lower than China’s association standard for the civil sanitary mask (≥90% filtration efficiency for 0.3 μm particles) (78) and much lower than ASTM F2100 standard (≥95% filtration efficiency for 0.1 μm particles) (79). By the end of 2021, there were still a significant number (36.07%) of students using masks with filtration efficiency lower than the national standard with an average filtration efficiency of 0.795 ± 0.119, which indicated that the serious inequality behind the quality of masks cannot be ignored. Moreover, during the COVID-19 outbreak (from December 2019 to June 15, 2020), the severe facemasks shortage in China created a boom in counterfeit production, resulting in the low-quality of masks flooding the market during that time (3). Of the masks used daily by students with a clear date of manufacture, about 11.43% were manufactured during the outbreak with a relatively low average filtration efficiency of 0.819 ± 0.152, compared to 0.903 ± 0.081 for masks produced after June 15, 2020.

Second, our empirical results presented in Figures 3A–C demonstrated that the better family economic status was associated with a better particle filtration efficiency rate which means better-quality masks, which strongly confirmed our hypothesis. The fractional regression results of the full sample (Figure 3A) indicated that the masks used daily by students from average families and students from rich families had 1.188 and 1.441 times higher particle filtration performance than the masks used by students from poor families, respectively. The model constructed based on the perspective of whether the masks are qualified or not (Figure 3D) also confirmed our hypothesis, that is, the better family economic status was, the greater the probability for students to use qualified masks. The masks used daily by students from ordinary families and wealthy families were 1.339 and 1.751 times more likely to be qualified than those used by students from poor families. What worth noticing is that the most significant difference remains between the poor and the rich.

Third, our empirical results presented in Figures 3E,F revealed that the students who had better family economic status were more likely to use the masks with individual packaging, and unique patterns and special designs, which also strongly confirmed our hypothesis. This may not only lead to a *de facto* inequality in the quality of the masks used daily by the poor and the rich, but also lead to inequality on a psychological level, that is, the poor can clearly perceive that the masks they use are low-cost and not fashionable enough.

Additionally, in terms of control variables, the regression results of Changsha sample indicated that the religious beliefs had significantly positive effect on masks’ filtration efficiency, whereas the party membership had significantly negative impact on the filtration performance of masks. The root cause of this may still lie in the family economic status. Previous studies have found that, in China, family economic conditions were positively correlated with religious beliefs (80), and students from better-off families were significantly less likely to join the CPC (81). Also, the regression results of the gender variable (see Figures 3E,F) demonstrated gender differences in mask selection, with female preferring masks with aesthetics design over individually packaged masks. Besides, the older students also had a greater preference for individually wrapped mask that was clean and hygienic.

Although nowadays most people consider the masks as a cheap everyday item (27), there are still a significant number of people who are price-sensitive and using the cheap but low-quality or even unqualified masks. This kind of inequality is not easily visible, because it is difficult for us to see the differences in the actual quality of different masks with naked eyes. The results of our research underscore the importance of the government providing sufficient free qualified personal protective equipment to everyone without discrimination during the pandemic. Rapid global warming highly increases the risk of future emerging infectious diseases (82). COVID-19 will not be the last infectious disease event of our time (83); we need to prepare for the next challenge with sufficient necessary medical resources, especially the qualified personal protective equipment.

This study had some limitations. Although our results were based on a much larger sample size compared with previous studies (38), the convenience sampling method may still cause a potential selection bias (1, 84). Besides, the limited air flow rate (2.83 L/min) of our particle counter CEM DT9881 may lead to some limitations in testing accuracy. Also, in an anonymous questionnaire, misrepresentation is inevitable, but we have minimized its impact on our study findings. Since we conducted the survey through teachers and counselors whom the students were familiar with, which means that we had the trusting endorsement from the teachers and counselors, so the students might be more willing to respond honestly to sensitive personal questions such as income level and family background.

## 5. Conclusion

Our analysis reveals the hidden socioeconomic inequality that exist behind the seemly cheap masks, and the family’s economic status was positively correlated with the quality of masks. The findings of this study have important implications for public health policy makers particularly those addressing future infectious disease control. In facing the challenges of future emerging infectious diseases, it is important for public health departments to address the inequity to ensure equal access to affordable qualified personal protection equipment.

To the best of our knowledge, this is the first article to evaluate the quality of masks in Mainland China based on a large set by testing their filtration efficiency for airborne particles, and the first empirical study to investigate the relationship between the quality of masks and family economic status.

There are still many questions that need to be further discussed. This study provides a starting point for future research. The following questions can be considered: Can this inequality phenomenon be observed in other general groups or other countries? In addition to the family’s economic status, do other socioeconomic factors such as occupation and social class also have a notable impact on the quality of masks? How does socioeconomic status influence people’s masking behavior, such as whether they choose to reuse the masks and the frequency of changing a mask?

## Data availability statement

The raw data supporting the conclusions of this article will be made available by the authors, without undue reservation.

## Ethics statement

The studies involving human participants were reviewed and approved by the School of Public Administration, Nanfang College · Guangzhou. Written informed consent from the participants’ legal guardian/next of kin was not required to participate in this study in accordance with the national legislation and the institutional requirements.

## Author contributions

LH conceived of the study, conducted the survey, evaluated the quality of masks, collected and analyzed the data, and prepared the manuscript draft. YiW provided comprehensive editing and refinement of the manuscript. BM, ZG, YuW, ZS, MH, HC, XM, and JX conducted the survey and collected the data. ML collected some additional mask samples and provided language editing of the manuscript. All authors contributed to manuscript revision, read, and approved the submitted version.

## Funding

This study was supported by the Young Innovative Talents Project of Colleges and Universities in Guangdong Province (2018WQNCX280) and School-level Research Projects of Nanfang College · Guangzhou (2021XK10).

## Conflict of interest

The authors declare that the research was conducted in the absence of any commercial or financial relationships that could be construed as a potential conflict of interest.

## Publisher’s note

All claims expressed in this article are solely those of the authors and do not necessarily represent those of their affiliated organizations, or those of the publisher, the editors and the reviewers. Any product that may be evaluated in this article, or claim that may be made by its manufacturer, is not guaranteed or endorsed by the publisher.
