# Supplementary material for: The hidden inequality: the disparities in the quality of daily use masks associated with family economic status
Source: Front Public Health. 2023 Jun 16;11:1163428. doi: 10.3389/fpubh.2023.1163428 (PMC10313325; doi:10.3389/fpubh.2023.1163428)
Supplement: Supplementary file 1 [file Data_Sheet_1.pdf]

Supplementary Material 2:

Rules for distinguishing between ordinary masks and masks with unique pattern or special design

We have a very simple and clear rule of distinction: masks like those shown in Figure 1 (including white, black and blue) are considered as ordinary masks, while the masks shown in Figure 2 is considered as masks with more fashionable and attractive unique pattern or special design. In fact, the difference between these two types of masks is obvious and can be easily distinguished.

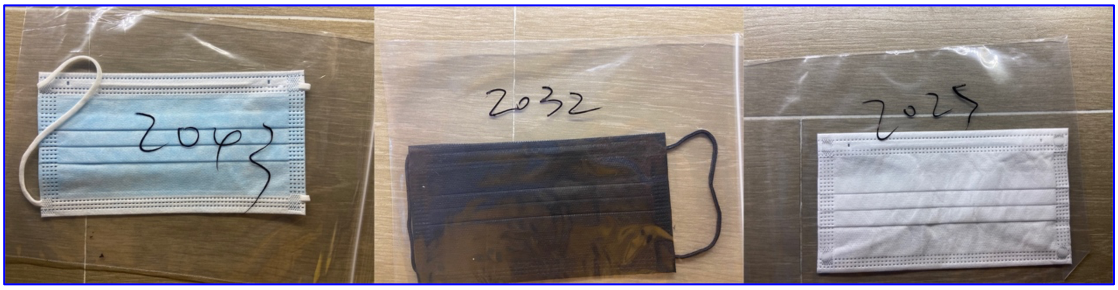

Figure 1. the photos of typical ordinary masks (including blue, balck and white)

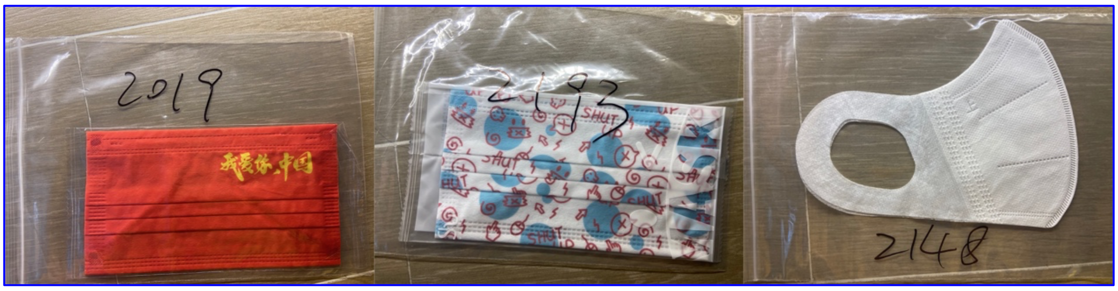

Figure 2. the photos of masks with more fashionable and attractive unique pattern or special design
